# Treatment with glatiramer acetate in APPswe/PS1dE9 mice at an early stage of Alzheimer’s disease prior to amyloid-beta deposition delays the disease’s pathological development and ameliorates cognitive decline

**DOI:** 10.3389/fnagi.2024.1267780

**Published:** 2024-01-30

**Authors:** Zengyong Huang, Zhuo Gong, Yongtai Lin, Fan Yang, Weiping Chen, Shaotong Xiang, Yuedong Huang, Hao Xiao, Shuwen Xu, Jinhai Duan

**Affiliations:** ^1^Eastern Department of Neurology, Guangdong Provincial People’s Hospital (Guangdong Academy of Medical Sciences), Guangdong Geriatrics Institute and Guangdong Cardiovascular Institute, Southern Medical University, Guangzhou, China; ^2^Shantou Central Hospital, Shantou, China; ^3^Zhongshan School of Medicine, Sun Yat-sen University, Guangzhou, China

**Keywords:** Alzheimer’s disease, amyloid-beta, neuroinflammation, regulatory T cells, glatiramer acetate

## Abstract

**Background:**

Alzheimer’s disease (AD) is characterized by neuroinflammation, which is frequently accompanied by immune system dysfunction. Although the mechanism of neurodegenerative lesions is unclear, various clinical trials have highlighted that early intervention in AD is crucial to the success of treatment. In order to explore the potential of immunotherapy in the early period of AD, the present study evaluated whether application of glatiramer acetate (GA), an immunomodulatory agent approved for remitting–relapsing multiple sclerosis (RRMS), in the early stages of AD prior to amyloid beta (Aβ) deposition altered the Aβ pathology and cognitive impairments in APPswe/PSEN1dE9 (APP/PS1) transgenic mice.

**Methods:**

We treated two cohorts of pre-depositing and amyloid-depositing (2- and 6-month-old) APP/PS1 mice with weekly-GA subcutaneous injection over a 12-week period. We then tested spatial learning and memory using the Morris water maze (MWM) and the Y maze. Immunohistochemistry staining was utilized to analyze Aβ burden in the brain as well as activated microglia. Furthermore, the inflammatory cytokine milieu within brains was estimated by quantitative real-time polymerase chain reaction, and the peripheral CD4^+^CD25^+^Foxp3^+^ regulatory T cells (Tregs) in the spleen were measured by flow cytometry.

**Results:**

We found that early GA administration reduced Aβ burden and ameliorated cognitive decline. Meanwhile, the immune microenvironment had changed in the brain, with an increase in the production of anti-inflammatory cytokines and a decrease in microglial activation. Interestingly, early GA administration also modulated the peripheral immune system through the amplification of Tregs in the spleen.

**Conclusion:**

Overall, our findings revealed that GA treatment might enhance the central and peripheral immune systems’ protective capabilities in the early stages of AD, eventually improving cognitive deficits. Our research supports the advantages of immunomodulatory treatments for AD at an early stage.

## 1 Introduction

Alzheimer’s disease (AD) is the most common form of dementia and one of the world’s leading causes of death. AD is a multifactorial neurodegenerative disease that involves a combination of the extracellular accumulation of beta-amyloid (Aβ) plaques, intracellular neurofibrillary tangles (NFTs), composed of hyperphosphorylated tau protein, chronic neuroinflammation, and reactive gliosis, ultimately leading to neuronal loss and declining cognition. However, effective treatments are still lacking (Authors, 2022; Karran and De Strooper, 2022). Fortunately, disease-modifying therapy (DMT) for AD is progressing through clinical trials; especially, Aβ-targeted monoclonal antibodies (mAb) (e.g., aducanumab, bapineuzumab, gantenerumab, solanezumab, and lecanemab), working on the amyloid hypothesis that an inability to clear Aβ contributes to the development and progression of AD, developed successfully and conducted in clinical trials (Cummings et al., 2022). These trials enrolled people with early AD, which includes people with mild cognitive impairment (MCI) or mild dementia due to AD. This implies that intervention at an early stage of AD is the key to promoting therapeutic success.

Burgeoning human data demonstrate that dysregulation of the immune system, including the innate and adaptive immune systems, is a cardinal feature of AD. Several studies indicate that dysregulation of inflammatory markers is evident in AD., which leads to an increased risk of the future development of all-cause dementia or AD (Swardfager et al., 2010; Koyama et al., 2013; Lai et al., 2017; Darweesh et al., 2018). Other evidence demonstrated that the peripheral homeostasis of T cells is partially altered in patients with AD, especially CD4^+^CD25^+^Foxp3^+^ regulatory T cells (Tregs). One study indicates that Tregs were more numerous in AD patients. Rosenkranz et al. (2007), while other studies found that the overall proportion of peripheral Tregs was lowered in MCI patients.in comparison to people with mild or severe AD dementia (Saresella et al., 2010; Le Page et al., 2017). Although strategies aimed at the depletion, amplification, or even transfer of Tregs are attracting increased attention as a potential treatment for Alzheimer’s disease, their precise contribution is complex and remains unclear (Yang et al., 2013; Baruch et al., 2015; Wang et al., 2015; Baek et al., 2016; Dansokho et al., 2016; Alves et al., 2017; Le Page et al., 2017).

**Figure 1:**
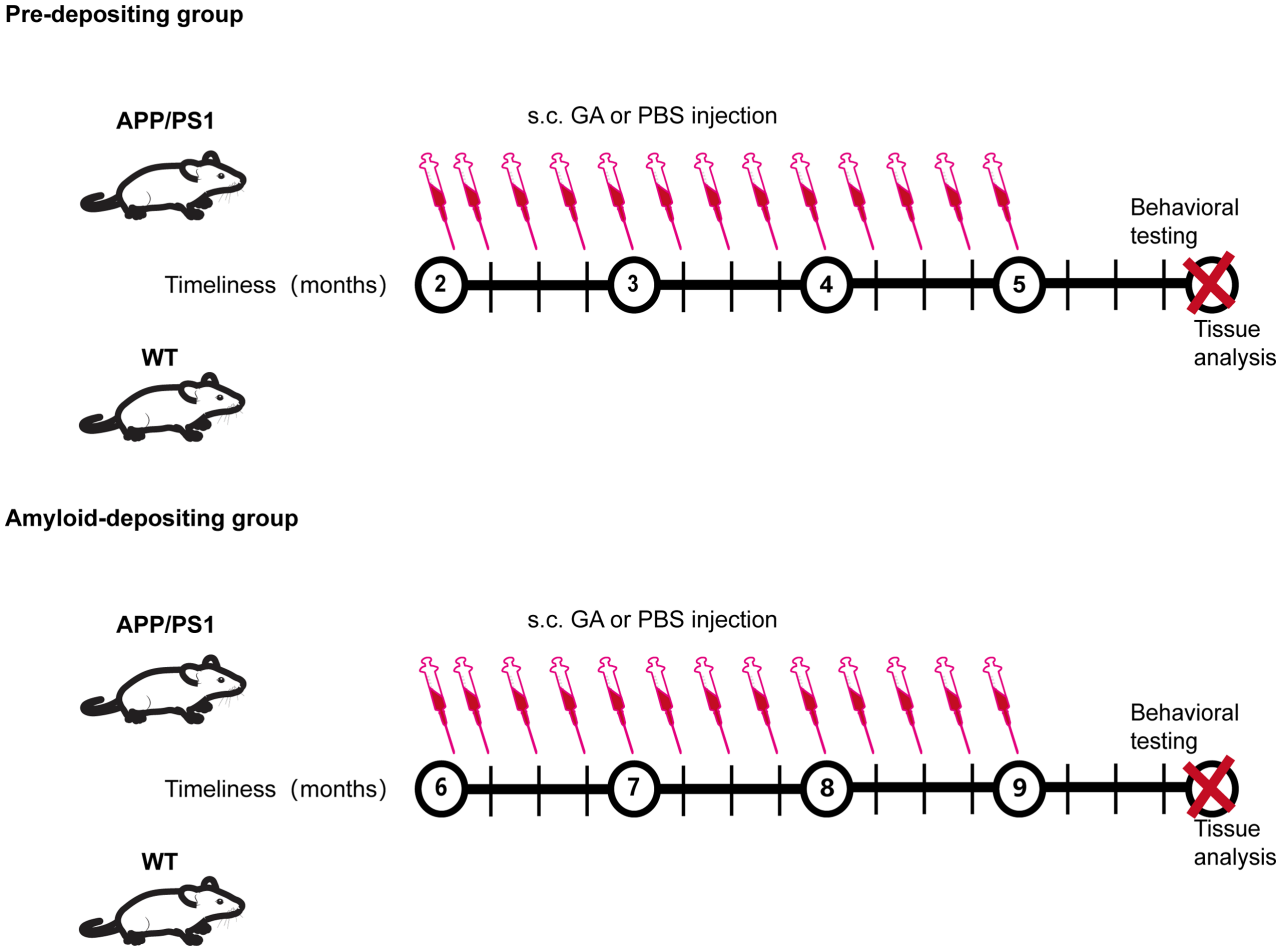
Experimental protocol.

Glatiramer acetate (GA), a drug approved by FDA for remitting–relapsing multiple sclerosis (RRMS), has been explored for its potential to reverse AD pathology and preserve memory and learning abilities in an AD mouse model (Butovsky et al., 2006, 2007; Bakalash et al., 2011; Koronyo et al., 2015; Rentsendorj et al., 2018; Doustar et al., 2020; Dionisio-Santos et al., 2021). As a mixture of four amino acid-based synthesized polypeptides, the method for GA administration in previous clinical and experimental research was subcutaneous injection due to the fact that most of a dose is slowly and sustainably degraded into smaller fragments in the subcutaneous department. In fact, several studies found that the therapeutic mechanisms of GA subcutaneous administration were attributed to reducing Tregs levels, enhancing recruitment of neuroprotective monocyte-derived macrophages in the brain, and rebalancing the levels of pro- and anti-inflammatory cytokines. While these encouraging therapeutic effects have been shown in early-symptomatic and in late-stage transgenic models of AD (7–20 months old), they have never previously been studied in an earlier stage prior to Aβ deposition. Given that a growing number of studies suggest that treatment intervention early in the process of the disease would provide better clinical benefits, we evaluated the impacts of GA immunomodulation in various stages of AD in APPswe/PSEN1dE9 (APP/PS1) transgenic mice, subcutaneously injected GA for 12 weeks at different time points (2- and 6-month-old), and, respectively, sacrificed the mice at 6 and 10 months of old (Figure 1). The results indicated that early subcutaneous injection of GA in APP/PS1 mice induced therapeutic benefits against AD, including expanding the frequency of peripheral Tregs, reducing the activation of microglia, and regulating the neuroinflammation by increasing the level of anti-inflammatory cytokines, which eventually delayed the impairment of cognitive function imposed by amyloid accumulation.

## 2 Materials and methods

### 2.1 Mice

Four-week-old and 5-month-old APP/PS1 mice, expressing a chimeric mouse/human amyloid precursor protein (Mo/HuAPP695swe) and a mutant human presenilin 1 (PS1-dE9), and their old-matched wide-type (WT) mice were purchased from the Laboratory Animal Centre of the Guangdong Medical Laboratory Animal Centre (Guangzhou, China). All mice were on the C57BL/6 congenic background. WT groups were used as ittermate controls. Only males were selected to minimize sex-specific differences. Before further treatment, the animals were housed in strictly scrutinized, specialized, and pathogen-free environments for 4 weeks. All animal research were conducted in accordance with the regulations of the Institutional Animal Care and Use Committee of Guangdong General Hospital.

#### 2.1.1 GA Vaccination

Each mouse (2 and 6 months old) received subcutaneous injections of 300 μg glatiramer acetate (Glpbio, GC14501) dissolved in 300 μL of phosphate-buffered saline (PBS) or PBS alone (control group) from experimental week 0 until week 12, twice during the first week and once per week thereafter (Figure 1).

### 2.2 Morris water maze

The experiments were performed in a 100-cm-diameter pool which is virtually divided into four quadrants. The water was made opaque with titanium dioxide, and its temperature was maintained at 21.5 ± 1.5°C. During the acquisition trail, mice were conducted twice a day for five consecutive days and learned how to find a hidden platform 1 cm below the water’s surface, near the center of the third quarter. The mice were placed in the maze facing the wall and allowed to swim for up to 60 s and remain on the platform for 5 s. If the mice failed to reach the platform in 60 s, they were physically placed on it and returned to their home cage after 15 s. The swimming distance and escape latency to reach the platform were measured. On day 6, the platform was taken away, and the mice had a single, 60s trial. An automated tracking system (TopScan™2.0, Clever Sys) recorded the data..

### 2.3 Y-maze

Three light-colored, opaque arms of a Y-shaped maze with a 120° angle between them are used for testing.. During the first trail, mice could freely explore two arms (‘start’ and ‘other’ arms) for 5 min while the third (‘novel’) arm was restricted by a divider. To eliminate preference-related bias, arm assignments were counterbalanced at random within each experimental group. Mice were placed back into home cage for 2 min. During the second trial, the test mouse from the first experiment was utilized again, this time in the distal portion of the same arm, and it was given 2 min to explore all three arms. The number of entries into each arm as well as the time spent in each arm were recorded by an automated tracking system (TopScan™2.0, Clever Sys). In order to eliminate any smell cues, the maze was meticulously cleaned with a 75% ethanol solution in between each exploration phase.

### 2.4 Quantitative enzyme linked immunosorbent assay of Aβ_1–40_ and Aβ_1–42_

The right brain tissue was homogenized and sonicated at 4°C in RIPA lysis buffer (Thermo Scientific™, cat. no. 89900) mixed with Mini Protease Inhibitor Cocktail (Roche, cat. no.04693124001). The supernatants were collected and quantified for the concentrations of total protein by using a bicinchoninic acid (BCA) protein reagent kit (Thermo Scientific™, cat. no. 23235). And Aβ_1-40_ and Aβ_1-42_ were individually measured by ELISA kits (R&D Systems, cat. no. DAB140B and cat. no. DAB142, respectively) according to the manufacturer’s protocols.

### 2.5 Immunohistochemistry and quantitative analyses

The left cerebral hemisphere was removed and post-fixed for 48 h at 4°C in 4% paraformaldehyde in 0.1 M PBS., and was subsequently cryoprotected overnight at 4°C in a 30% sucrose/PBS solution. Free-floating 30-μm thick coronal brain sections were cut on a freezing microtome (Leica) and stored in 0.1% NaN3 and PBS. Prior to staining, the tissue sections were washed three times for 5 min in PBS and blocked for 60 min at room temperature with blocking buffer (10% donkey serum, 0.3% Triton X-100, and PBS), followed by overnight incubation with the primary antibody in blocking buffer. After washing three times in PBS, the sections were incubated with Alexa Fluor 488 donkey anti-goat or Alexa Fluor 594 donkey anti-mouse antibodies (1:1000, Invitrogen, cat. no. A-11055 and cat. no. SA5-10168) for 90 min at room temperature and then washed again. Sections were covered with Citoglas® (cat. no. 188105w) mounting medium with 4′,6-diamidino-2-phenylindole (DAPI) (Solarbio, cat. no. S2100). The following primary antibodies were used with their respective concentrations: mouse anti-Aβ (1:1000, Novus, cat. no. NBP2-13075), goat anti-Iba1 (1:500, Novus, cat. no. NB100-1028).

For the image analysis, sections were imaged under a LSM900 confocal laser scanning microscope (Zeiss). Six brain sections with a defined distance (10 coronal sections spaced 200 μm) apart from each other were imaged. ImageJ software (NIH) was used to quantify the staining areas in each image’s region of interest. For the quantification of Aβ burden, six random images of the cortex and one image for the whole hippocampus were evaluated. The percentage of Aβ staining was determined in relation to the total area of the analyzed region. To quantify the microglia recruitment towards Aβ burden, six random images with the same region of interest (ROI) were collected at 20× magnification in similar regions of the cortex and hippocampus. The ROI was delineated as the surrounding area for each Aβ deposit. The ratio of the Aβ staining area to the double-labelled cells’ positive areas for Iba1 and DAPI was measured. All data was analyzed in a double-blind manner.

### 2.6 Flow cytometry

For regulatory T cell staining, spleens were crushed using a syringe’s plunger and treated with ACK (ammonium chloride potassium)-lysing buffer to remove erythrocytes and generate a single cell suspension. All cells were first blocked with anti-Fc CD16/32 (1:100, eBioscience) to avoid non-specific staining. FITC-conjugated anti-CD4 (11–0041-82) and eFluor 450-conjugated anti-CD25 (48–0251-82) were used for cell-surface staining. In order to intranuclear staining, cells were fixed and permeabilized using the Foxp3 Fix/Perm Buffer Set (00–5, 523-00), and then incubated with APC-conjugated anti-FOXP3 (17–5, 773-82). All the fluorochrome-labelled monoclonal antibodies listed above were obtained from eBioscience and employed according to the manufacturer’s instructions. Fluorescence data were collected on a Beckman CytoFlex and analyzed using Flowjo software,

### 2.7 Quantitative real-time polymerase chain reaction

Total RNA of the cerebrum was extracted with TRIzol™ Reagent (Invitrogen™, cat. no. 15596026). PrimeScript™ RT reagent Kit (Takara, RR037A), and then was reverse transcribed using the PrimeScript™ RT reagent Kit (Takara, RR037A). qRT-PCR analysis was conducted using TB Green® Premix Ex Taq™ II (Tli RNaseH Plus) (Takara, RR820A). β-Actin was chosen as a reference (housekeeping) gene. Amplification conditions were: 95°C for 30s, then 39 cycles at 95°C for 5 s and 60°C for 30s. At the end of the amplification cycles, a melting curve was produced to assess the reaction’s specificity. The CFX96 Real-Time PCR Detection System (Bio-Rad) was used to perform and analyze all qRT-PCR experiments. For the experiments, the following primers were used:

β-actin: forward primer 5’-TTCCAGCCTTCCTTCTTGGGT-3′ and reverse primer 5’-CTTTACGGATGTCAACGTCACAC-3′;

TNF-α: forward primer 5’-CCCAAAGGGATGAGAAGTTCC-3′ and reverse primer 5’-GCTACAGGCTTGTCACTCGAA-3′;

IL-6: forward primer 5’-CGGCCTTCCCTACTTCACAA-3′ and reverse primer 5’-TGCCATTGCACAACTCTTTTC-3′;

IL-1β: forward primer 5’-TGCCACCTTTTGACAGTGATG-3′ and reverse primer 5’-GTGCTGCTGCGAGATTTGAA-3′;

TGF-β1: forward primer 5’-CTTCAATACGTCAGACATTCGGG-3′ and reverse primer 5’-GTAACGCCAGGAATTGTTGCTA-3′;

IL-4: forward primer 5’-CCCCAGCTAGTTGTCATCCTG-3′ and reverse primer 5’-CAAGTGATTTTTGTCGCATCCG-3′;

### 2.8 Statistical analysis

Statistical analyses were performed using GraphPad Prism version 9 (GraphPad). The escape latency data were analyzed using a two-way ANOVA followed by Tukey’s multiple comparison. Significant differences were determined using either Student’s t-test for two-group comparisons or one-way ANOVA followed by an LSD *post hoc* comparison between more than two groups. Data were expressed as the mean ± standard error of the mean (SEM), and *P* < 0.05 was regarded as statistically significant.

## 3 Results

### 3.1 GA ameliorated learning and memory deficits in APP/PS1 mice

**Figure 2:**
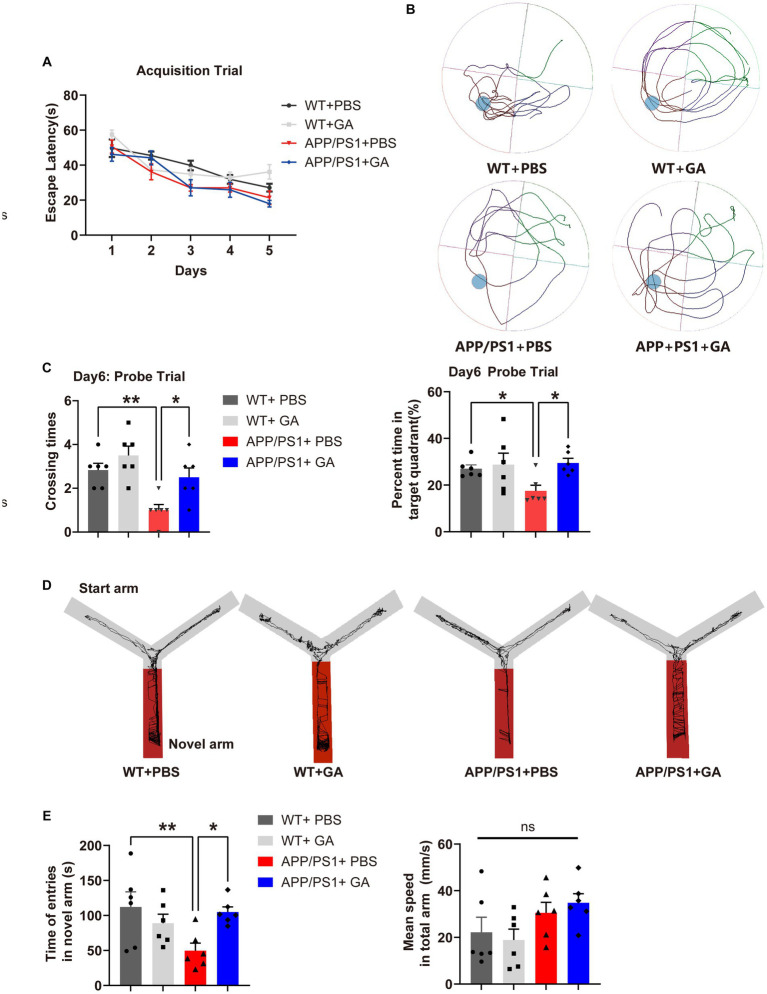
Impact of GA treatment on the spatial learning and memory of APP/PS1 mice receiving intervention from the pre-deposition period. **(A–C)** MWM test was conducted to assess the spatial learning and memory of mice. **(A)** No significant difference in the escape latency was shown between APP/PS1 mice and WT mice in the acquisition trail [two-way ANOVA followed by Tukey’s multiple comparison test, treatment-time *F*(12,80) = 0.8134, *p* = 0.636]. **(B)** Representative traces during the spatial probe trial. **(C)** The crossing times and the percent time in the target quadrant in the probe trial by APP/PS1 mice were significantly lower compared to WT mice [*F*(3, 20) = 1.833, *p* = 0.0019; *F*(3, 20) = 9.527, *p* = 0.0371], while they were significantly improved after receiving GA injection [*F*(3, 20) = −1.5, *p* = 0.0085; *F*(3, 20) = −11.98, *p* = 0.0108]. **(D,E)** Evaluation of the spatial memory of mice by Y maze text. **(D)** Representative traces in each arm during the spatial novelty recognition trail. **(E)** APP/PS1 mice spent less time exploring the novel arm during the spatial novelty recognition trail than WT mice [*F*(3, 20) = −62.29, *p* = 0.0055], but GA treatment greatly increased the duration [*F*(3, 20) = −55.13, *p* = 0.0122]. Importantly, this tendency was not caused by a variation in speed between the groups [*F*(3, 20) = −2.211, *p* = 0.1184]. All data were analyzed in the PBS-treated WT mice, GA-treated WT mice, PBS-treated APP/PS1 mice, and GA-treated APP/PS1 mice (6-month-old, mean ± SEM, *n* = 6 per group, one-way ANOVA, and LSD *post hoc* analysis; *p < 0.05, **p < 0.01).

Previous research has shown that APP/PS1 mice develop cerebral amyloidosis as early as 2–3 months of old, and exhibit obvious Aβ pathologies and memory deficits at 6–8 months old (Kurt et al., 2003; Yan et al., 2009). To examine the possible therapeutic value of GA for disease progression in APP/PS1 mice, we therefore analyzed two cohorts of pre-depositing and amyloid-depositing (2- and 6-month-old) mice utilizing weekly GA administration for 12 weeks (Figure 1). The Morris Water Maze (MWM) task was used to assess the cognitive performance of APP/PS1 mice 1 month following the last GA administration. In the pre-depositing 2-month-old group, we found that there was no significant difference between APP/PS1 mice and WT mice in spending time searching for the platform (escape latency) (Figure 2A), indicating that cognition in terms of spatial learning was not yet impaired in PBS-treated APP/PS1 mice compared to WT mice. However, during the probing trial used to evaluate the preservation of spatial memory, target quadrant time and frequency were decreased in APP/PS1 mice. Than WT mice. In addition, GA-treated APP/PS1 mice passed across the platform more frequently and spent more time in the target quadrant compared with PBS-treated APP/PS1 mice (Figures 2B,C). In contrast, in the amyloid-depositing 6-month-old group, when compared to PBS-treated WT mice, both PBS-treated and GA-treated APP/PS1 mice showed significant impairment in spatial learning and memory ability, as evidenced by more time in escape latency, either less time spent in the target quadrant or crossovers during the probe trail (Supplementary Data). We also examined the spatial memory recovery of GA-treated pre-depositing 2-month-old APP/PS1 mice with the Y-maze test. We discovered that GA treatment significantly lengthened the exploration time of APP/PS1 mice in the novel arm during the spatial novelty recognition trail (Figures 2D,E). These findings suggested that early GA treatment improved APP/PS1 mice’s spatial memory.

All male mice were separated into two age groups: the pre-depositing (2-month-old) group and the amyloid-depositing (6-month-old) group. They were subcutaneously (s.c.) injected with GA or PBS for 12 weeks, twice during the first week and once per week thereafter. Behavior tests were carried out 1 months following the last injection to assess the spatial learning and memory abilities of the mice. Then, spleen tissues were gathered for the FCM analysis, and brain tissues were collected for IHC, qRT-PCR, and ELISA analysis.

### 3.2 GA treatment alleviated A**β** burden and A**β** levels in the cortex and hippocampus of APP/PS1 mice receiving intervention from the pre-deposition period

**Figure 3:**
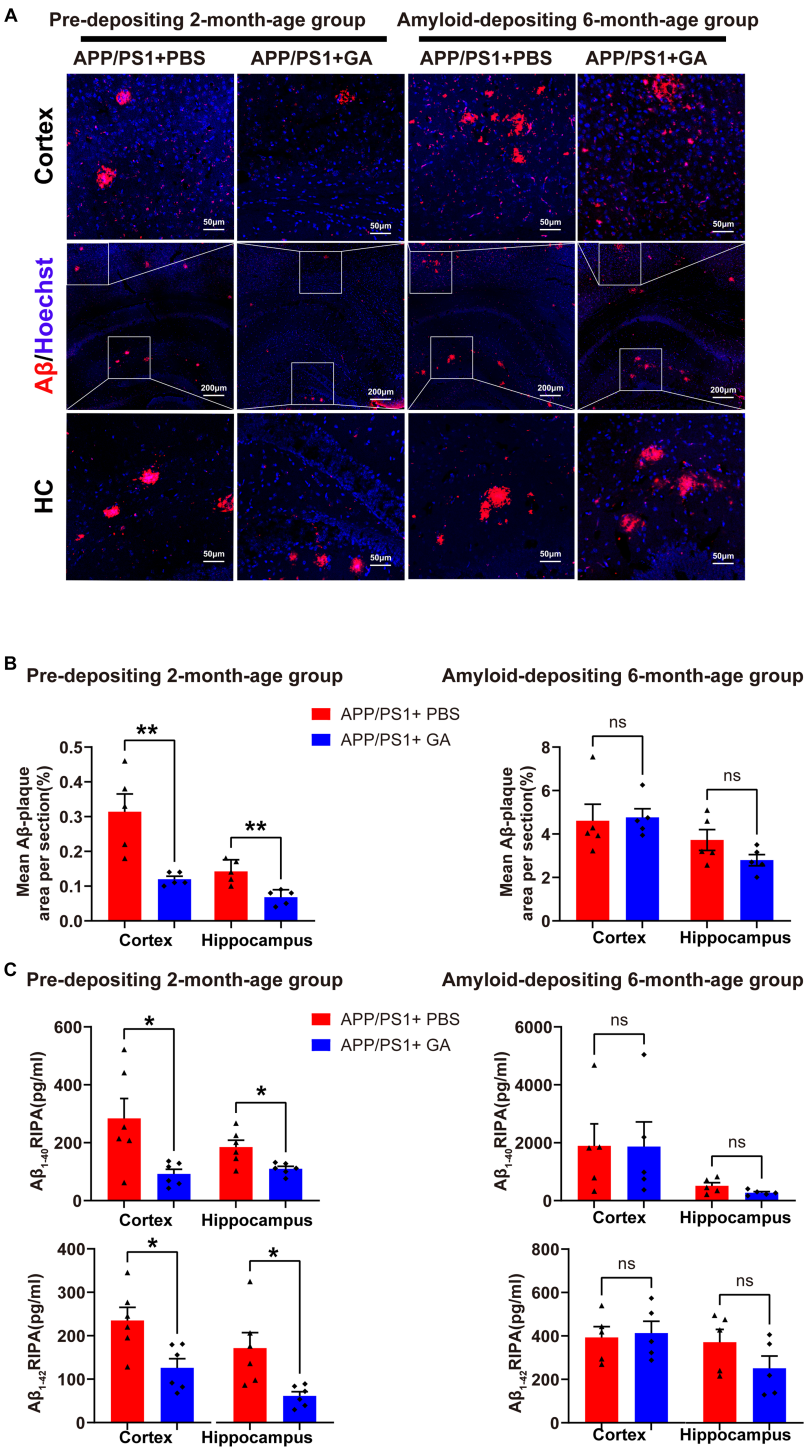
GA treatment attenuates the Aβ burden in APP/PS1 mice receiving intervention from the pre-deposition period. **(A)** Representative microscopy images of the cortex and hippocampus of APP/PS1 mice, stained for Aβ plaques (red) and with DAPI (blue). **(B)** Quantification of the percentage of the mean area of Aβ plaques in each cerebral section (*n* = 6 per group) showed that GA administration effectively decreased Aβ plaque deposition in the pre-depositing 2-month-age group in both the cortex and hippocampus (*t*_8_ = 3.729, *p* = 0.0058; *t*_8_ = 3.729, *p* = 0.0032), but the difference between PBS-treated and GA-treated APP/PS1 mice in the amyloid-depositing 6-month-age group was not significant (*t*_8_ = 0.177, *p* = 0.8637; *t*_8_ = 1.71, *p* = 0.1249). **(C)** *ELISA* analysis of the Aβ_1–40_ and Aβ_1–42_ from the cerebral tissue extracted with RIPA (*n* = 5 per group) demonstrated that both of them were reduced in the cortex and hippocampus of GA-treated APP/PS1 mice in the pre-depositing 2-month-age group, (cortex – Aβ_1–40_: *t*_10_ = 2.707, *p* = 0.0221; hippocampus – Aβ_1-40_: *t*_10_ = 2.961, *p* = 0.0143; cortex – Aβ_1–42_: *t*_10_ = 2.971, *p* = 0.0140; hippocampus – Aβ_1–42_: *t*_10_ = 2.954, *p* = 0.0144), while differences among PBS-treated or GA-treated APP/PS1 mice in the amyloid-depositing 6-month-age group were not found significant (cortex – Aβ_1–40_; *t*_8_ = 0.02438, *p* = 0.9811; hippocampus – Aβ_1-40_: *t*_8_ = 1.974, *p* = 0.0839; cortex – Aβ_1-42_: *t*_8_ = 0.267, *p* = 0.7962; hippocampus – Aβ_1–42_; *t*_8_ = 1.457, *p* = 0.0.1832). ELISA assays were done in five biological replicates with technical triplicates each. (The pre-depositing group: 6-month-old; the amyloid-depositing group: 10-month-old; mean ± SEM, Student’s *t*-test, and LSD *post hoc* analysis; *p < 0.05, **p < 0.01).

Following the behavioral test, we investigated the impact of early GA treatment on Aβ pathology in the hippocampus and cerebral cortex of APP/PS1 mice. Immunohistochemistry (IHC) revealed typical Aβ plaques in the hippocampus and cortex of both the pre-depositing and the amyloid-depositing groups. However, only the pre-depositing APP/PS1 mice that received GA injections had a substantially lower mean area with Aβ plaques in the cortex or hippocampus compared to control-treated animals (Figures 3A,B). The outcomes were in line with the quantification analysis of Aβ_1–40_ and Aβ_1–42_ extracted from the cortex and hippocampus of the APP/PS1 mice by RIPA using ELISA (Figure 3C). Collectively, our observations imply that early GA intervention reduces Aβ levels in APP/PS1 mice, which may be crucial to the observed cognitive improvement.

### 3.3 GA treatment attenuated microgliosis and increased anti-inflammatory cytokine levels in APP/PS1 mice receiving intervention from the pre-deposition period

**Figure 4:**
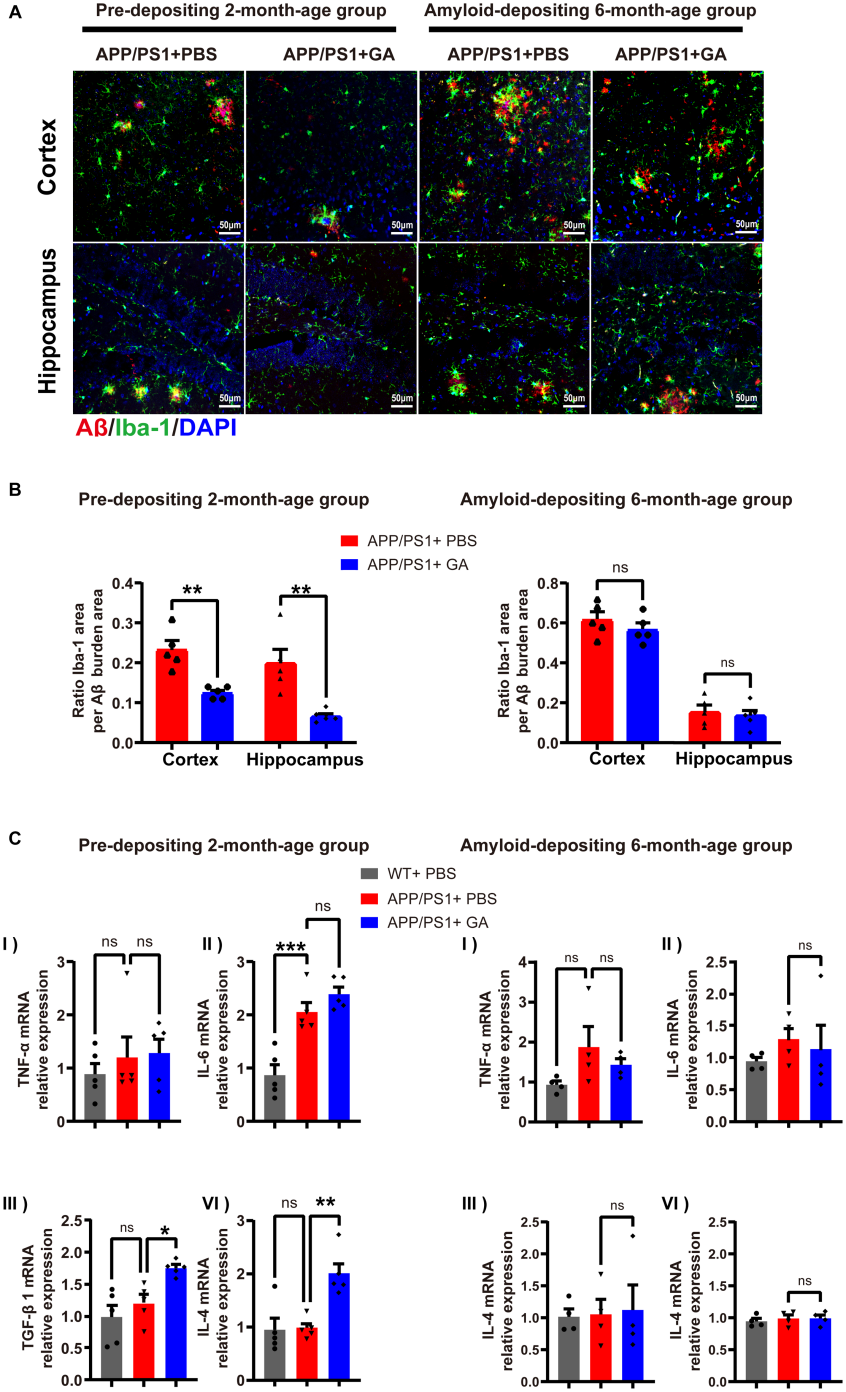
GA therapy reduced microglia activation and elevated anti-inflammatory cytokine levels in APP/PS1 mice receiving intervention from the pre-deposition period. (**A)** Representative microscopy images of Aβ (red) colocalized with Iba-1-immunoreactive microglia (green) and DAPI (blue) in the cortex and hippocampus of APP/PS1 mice. **(B)** Quantification of the ratio Iba-1+ immunoreactive area to Aβ burden area in the cortex and hippocampus: (left) in the young age group, the reduction in APP/PS1 mice received GA injections was more obvious than that in those treated with PBS (*t*_8_ = 4.682, *p* = 0.0016; *t*_8_ = 3.826, *p* = 0.005); (light) while in the amyloid-depositing stage of APP/PS1 mice, there was no difference between GA-treated and PBS-treated groups (*t*_8_ = 1.034, *p* = 0.3314，*t*_8_ = 0.4678, *p* = 0.6524) (*n* = 5 per group, Student’s *t*-test, and LSD *post hoc* analysis). **(C)** qRT-PCR examination of the mRNA expression levels of the pro-inflammatory (TNF-α and IL-6) and anti-inflammatory (TGF-β1 and IL-4) cytokines in brains (one-way ANOVA, and LSD *post hoc* analysis). In the pre-depositing 2-month-old group, the mRNA expression levels of IL-6 **(C-II)** were increased in both GA-treated and PBS-treated APP/PS1 mice compared to the WT mice [*F*(2, 12) = −1.184, *p* = 0.0004, *F*(2, 12) = −1.522, *p* < 0.0001]. TNF-α **(C-II)** seemed to be on the same increasing trend. While increased levels of TGF-1β **(C-III)** and IL-4 **(C-IV)** mRNA were observed in APP/PS1 mice after GA therapy [TGF-1β: *F*(2, 12) = −0.546, *p* = 0.0146; IL-4: *F*(2, 12) = −1.016, *p* = 0.0013]. qRT-PCR assays were done in five biological replicates with technical triplicates each. (The pre-depositing group: 6-month-old, *n* = 4; the amyloid-depositing group 10-month-old, *n* = 5; mean ± SEM, *p < 0.05, **p < 0.01).

Given the chronic neuroinflammation induced by microglia surrounding Aβ plaques is tightly linked to AD progression (Heneka et al., 2015), we investigated the potential inflammatory regulation of GA on APP/PS1 mice. To assess the microglial reactivity in the cortex and hippocampus, we performed Iba-1 immunostaining of brain sections and confirmed that the GA treatment could significantly reduce the Iba-1+ immunoreactive microglia around Aβ plaques in the pre-depositing APP/PS1 mice group, but whether the amyloid-depositing 6-month-old group received GA injection or not made no difference (Figures 4A,B). In addition, qRT-PCR analysis of the cerebral inflammatory cytokine milieu indicated increased mRNA expression levels of the pro-inflammatory IL-6 in both GA-treated and PBS-treated APP/PS1 mice relative to the WT mice among the pre-depositing 2-month group. TNF-α mRNA expression levels also appear to be growing. (Figures 4C,I-III). Interestingly, the anti-inflammatory mRNA expression levels of TGF-1 and IL-4 were considerably increased in APP/PS1 mice following early GA intervention starting at 2 months of old (Figures 4CIII,IV), consistent with the above results, implying that GA is involved in modulating the immune microenvironment in AD brains by attenuating microglia activation and increasing anti-inflammatory cytokine levels.

### 3.4 Effects of GA on CD4^+^CD25^+^Foxp3^+^ regulatory T cell responses in APP/PS1 mice

**Figure 5:**
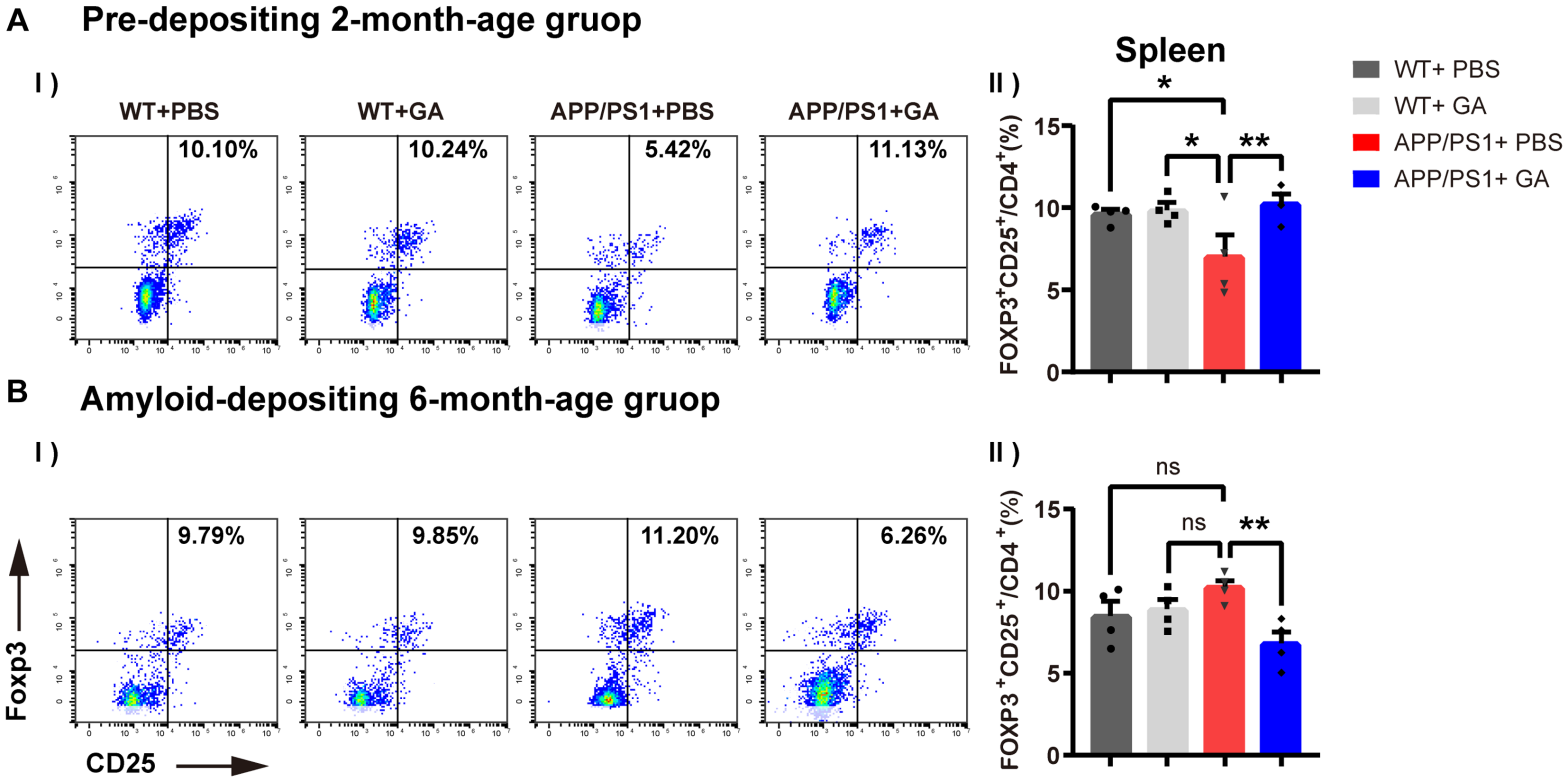
Effects of GA on CD4^+^CD25^+^Foxp3^+^ regulatory T cell responses in APP/PS1 mice. **(A-I,II)** Representative flow cytometry plots of Tregs. **(B-I,II)** Quantitative analysis of the proportions of CD4^+^CD25^+^Foxp3^+^ Tregs in the spleen indicated that the frequencies of Tregs in young APP/PS1 mice **(B-I)** were lower than those of their wild-type littermates [*F*(3, 12) = 2.586, *p* = 0.0324], and were able to amply via GA treatment [*F*(3, 12) = −3.268, *p* = 0.0099]. But in the stage of amyloid-depositing, GA administration showed a reduction in the Tregs population of APP/PS1 mice [*F*(3, 12) = 3.433, *p* = 0.0036]. The assays were done in four biological replicates with technical doubles each. (The pre-depositing group: 6-month-old; the amyloid-depositing group 10-month-old; mean ± SEM, *n* = 4 per group, one-way ANOVA and LSD *post hoc* analysis, **p < 0.01).

Previous research has shown that GA increases the frequency and activity of Tregs as well as the expression of FOXP3, a gene involved in immune system regulation (Hong et al., 2005; Weber et al., 2007). In addition, emerging evidence suggests Tregs play a neuroprotective role by suppressing inflammation in the central nervous and peripheral immune system (Sakaguchi et al., 2008; Kramer et al., 2019). Therefore, following the last GA injection, we further analyzed CD4^+^CD25^+^Foxp3^+^ regulatory T cell frequencies in the spleen of pre-depositing and amyloid-depositing mice, including GA-treated APP/PS1 mice, PBS-treated APP/PS1 mice, and old-matched littermate control mice utilizing flow cytometry. We found that in PBS-treated APP/PS1 mice, the young group showed significantly diminished frequencies of Tregs relative to their wild-type littermates (Figure 5), but an increasing trend with age was observed. Intriguingly, weekly GA administration in APP/PS1 mice before the onset of amyloid-deposition played a promoting role in Tregs expansion, whereas after the onset of amyloid-deposition, GA administration showed a depletion in the Tregs population. Collectively, the different timing of GA intervention in APP/PS1 mice appeared to have different efficacies in Treg frequencies.

## 4 Discussion

In this study, we showed that GA therapy may help the amplification of Tregs to reduce microglia recruitment and elevate protective anti-inflammatory cytokines at early stages of AD, thereby altering amyloid pathology and improving cognitive performance.

GA has been used for a commonly prescribed disease-modifying therapy for RRMS for more than two decades. Although the mechanism of action of GA is still not fully understood, recent preclinical research using GA found its potential neuroprotective effects in neurodegenerative disease models, such as AD, Parkinson’s disease (PD), and Huntington’s disease (HD) (Kasindi et al., 2022). Studies examining in symptomatic transgenic mouse models of AD, including 5xFAD, 3xFAD or APP_SWE_/PS1_ΔE9_, have found that weekly GA vaccination for 8 weeks reversed plaque pathology and prevented cognitive decline (Butovsky et al., 2006; Bakalash et al., 2011; Baruch et al., 2015; Koronyo et al., 2015; Dansokho et al., 2016; Doustar et al., 2020; Dionisio-Santos et al., 2021). Nevertheless, the potential of long-term GA treatment in young mice with no amyloid pathology development has yet to be investigated. Our study found that early GA treatment for 12 weeks in the young APP/PS1 mice without amyloid depositing attenuated amyloid pathology and improved spatial learning and memory in the MWM and Y-maze tests. Interestingly, in contrast to the positive impact of early GA treatment, our results showed that APP/PS1 mice following GA injections in a pre-symptomatic stage of amyloid-deposition did not exhibit any beneficial effect on either cognitive performance or plaque load. One possible reason for this discrepancy could be that the impact of GA in AD depends on multifactor influences, such as varying session duration, drug frequency, or disease states. For instance, some research evaluated the effects of administering GA to transgenic mice with AD on a daily versus weekly basis and found quite different results; weekly treatment showed an advantage, while daily GA injections even had a mildly negative effect on plaque load and cognitive function (Baruch et al., 2015). Additionally, certain studies examining the effects of daily versus biweekly GA administration in Amyotrophic Lateral Sclerosis (ALS) patients found that daily treatment improve Th2 cytokines, whereas twice-weekly treatment was linked to an increase in Th1 cytokines (Mosley et al., 2007). These results highlight the need for further investigation into the mechanism of action of GA at multiple dosages, session durations, and disease states.

It is important to note that growing evidence from genome-wide association studies (GWAS) has shown that a variety of AD risk genes are involved in inflammation signaling that influences the development and course of the disease (Marioni et al., 2018; Jansen et al., 2019), supporting the notion that sustained inflammation caused by abnormally folded Aβ and tau proteins accumulation plays a crucial role in the pathophysiology of Alzheimer’s disease. Considering the Tregs’ pivotal role in maintaining self-tolerance and in limiting excessive immunological reactions harmful to the host (Sakaguchi et al., 2008), emerging strategies attempt to examine the potential of Tregs regulation as a therapy in the progression of animal models of AD. In our study, APP/PS1 mice that received GA injections as young as 2 months old showed a significant amplification in the frequency of Tregs and an increase in the expression of anti-inflammatory cytokines compared to those in APP/PS1 mice treated with PBS. Consistent with our observation, (Dansokho et al., 2016) showed that using the 4–6-week-old AD mouse model (APP/PS1), the amplification of Tregs by peripheral chronic low-dose IL-2 administration restored cognitive functions, whereas the transient depletion of Tregs accelerated the onset of cognitive decline (Dansokho et al., 2016). Baek et al. (2016) showed that the adoptive transfer of Tregs into 4-month-old 3xTg-AD mice improved cognitive function and reduced Aβ burdens, whereas similarly, the depletion of Tregs in 4-week-old 3xTg-AD mice resulted in aggravation of the disease progression (Baek et al., 2016). Furthermore, our studies revealed that GA might have dichotomous effects on Tregs in APP/PS1 mice: the amplification of Tregs in the pre-depositing stage and the depletion of Tregs in the amyloid-depositing stage. Baruch et al. (2015) also found the similar result that weekly GA treatment in 6-month-old 5XFAD mice reduced splenocyte Tregs levels. However, differing from our finding that no effective impact of Tregs’ depletion on alleviating the development of AD was observed in amyloid-depositing APP/PS1 mice, they suggested that the depletion of Tregs induced the activation of the brain’s choroid plexus, followed by amyloid-β plaque clearance, mitigation of the neuroinflammatory response, as well as a reversal of cognitive deterioration (Baruch et al., 2015). Apparently, Tregs play an important role in the processing of AD, yet their specific contribution is complex and uncertain so far. We considered that Tregs might have had a beneficial role in the early stages of AD, while they become dysfunctional with the progression of the disease. This hypothesis is consistent with the previous studies that have demonstrated that anti-inflammatory and immunosuppressive Treg function was impaired in AD patients (Ciccocioppo et al., 2019; Faridar et al., 2020) and is supported by the finding that *ex vivo* expanded Tregs restored their immunomodulatory function and alleviated AD pathology in a preclinical model of AD (Faridar et al., 2022). Furthermore, a recent study of immune cells revealed that Tregs reside in the brain (Korin et al., 2017, 2018). Under neuroinflammatory circumstances, the central and peripheral immune responses result in Treg infiltration into the brain (Schetters et al., 2017; Machhi et al., 2020), where they exert a direct anti-inflammatory effect (Xie et al., 2015; Machhi et al., 2020). But as the disease progresses, the function of the meningeal lymphatics is impaired, resulting in the blockage of their infiltration (Da Mesquita et al., 2018). Perhaps it is the double hit of dysfunction of Tregs and meningeal lymphatics that leads to the complicating results of the study in the late period of AD. Of note, as more recent studies demonstrated that Aβ antigen-specific Tregs were more effective at alleviating neuroinflammation and recovering cognitive impairment in a model mouse of AD in contrast to polyclonal Tregs (Yang et al., 2022; Jung et al., 2023), the roles that different Treg subtypes play should not be ignored. Altogether, these results point to the involvement of Tregs in the pathophysiology of AD, but their actual contribution and phenotype in different disease stages need to be further studied.

Previous research has found that Aβ plaques and activated microglia colocalize in the brains of AD patients, suggesting an interaction between these two key pathogenic features of AD (McGeer et al., 1987; Tooyama et al., 1990). Likewise, our study found that GA-treated ahead of amyloid deposition not only significantly reduced microglia activation but also alleviated Aβ burden in APP/PS1 mice. However, the arguments about whether the disease is brought on by microglia surrounding the plaques or whether AD pathology triggers a subsequent reaction in the microglia are still ongoing. In a recent study, in order to investigate the role of microglia in the early phases of AD plaque formation, the researchers designed and synthesized a unique brain-penetrant CSF1R inhibitor (PLX5622) allowing for the sustained and specific elimination of microglia prior to and during the period of plaque formation in the 5xFAD mice. They found that, except in areas where microglia are still present, plaques do not develop in the parenchymal of the brain after microglial removal, suggesting that microglia play key roles in triggering plaque pathogenesis (Spangenberg et al., 2019). Intriguingly, microglia might not only be involved in the launch of Aβ formation but may also contribute to the seeding and spreading of Aβ pathology. For instance, microglia might excrete ASC specks, an adaptor protein apoptosis-associated speck-like protein containing a CARD (ASC), after the NACHT-,LRR- and pyrin (PYD)-domain-containing protein 3 (NLRP3) inflammasome activation induced by Aβ, causing them to bind rapidly to Aβ and promote the production of Aβ oligomers and aggregates (Venegas et al., 2017). Alternatively, excessive accumulation of Aβ within microglial lysosomes might cause cellular death, which could lead to the release of Aβ aggregates at the location of microglial death, resulting to Aβ expansion (Baik et al., 2016). Considering the above, the effectiveness of early GA intervention in AD may be attributed to preventing the formation of Aβ plaque by reducing microglia activation. Notably, transcriptomic studies in AD mouse models identified that a special microglial phenotype, termed disease associated microglia (DAM), which is close to Aβ plaques, participate in Aβ clearance, suggesting their protective role in AD (Keren-Shaul et al., 2017). Regrettably, considering the limitation of our study is the lack of identification of microglia subsets, the mechanism linking different microglia subsets to GA and AD pathology in the transgenic mouse model is required for further study.

## 5 Conclusion

This work substantiated that weekly GA-administration for 12 weeks during the early stage of AD prior to amyloid deposition improved cognitive performance and alleviated Aβ pathology in APP/PS1 mice through its effect on the amplification of peripheral Tregs, reduction of microglia activation, and increased the level of anti-inflammatory cytokines. But current results lacked proof of mutual causality about the relationship between Aβ deposition and central and peripheral immune or inflammatory changes. We hope further investigation will find a hypothesized mechanism, especially their actual contribution and phenotype in different disease stages. Taken together, GA administration in the early stages of AD may be a highly valuable therapeutic strategy for halting or delaying AD disease development.

## Data availability statement

The raw data supporting the conclusions of this article will be made available by the authors, without undue reservation.

## Ethics statement

The animal study was approved by the Institutional Animal Care and Use Committee of Guangdong General Hospital. The study was conducted in accordance with the local legislation and institutional requirements.

## Author contributions

ZH: Formal analysis, Investigation, Methodology, Visualization, Writing – original draft. ZG: Formal analysis, Investigation, Writing – original draft. YL: Investigation, Writing – original draft. FY: Investigation, Writing – original draft. WC: Formal analysis, Writing – review & editing. ShaX: Formal analysis, Writing – original draft. YH: Methodology, Writing – review & editing. HX: Methodology, Writing – review & editing. ShuX: Supervision, Writing – review & editing. JD: Conceptualization, Funding acquisition, Methodology, Project administration, Supervision, Writing – original draft, Writing – review & editing.
