# Supplementary material for: Treatment with glatiramer acetate in APPswe/PS1dE9 mice at an early stage of Alzheimer’s disease prior to amyloid-beta deposition delays the disease’s pathological development and ameliorates cognitive decline
Source: Front Aging Neurosci. 2024 Jan 30;16:1267780. doi: 10.3389/fnagi.2024.1267780 (PMC10861656; doi:10.3389/fnagi.2024.1267780)
Supplement: Supplementary file 1 [file Image_1.pdf]

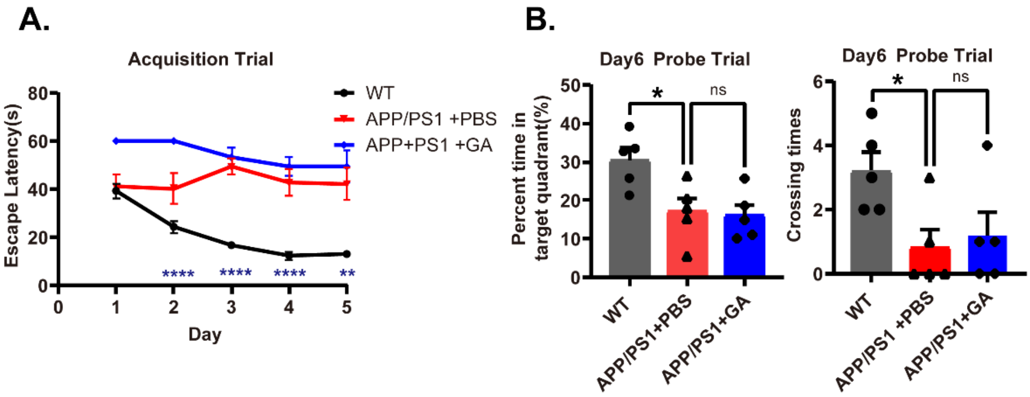

Supplementary Data. GA administration failed to restore cognitive functions in amyloid-deposition (6-month-old) APP/PS1 mice.

MWM test was conducted to assess the spatial learning and memory of mice. In the acquisition trail (**A**), PBS-treated and GA-treated APP/PS1 mice both spent more time in escape latency than WT mice, but the difference between them was not significant (two-way ANOVA followed by Tukey's multiple comparison test, treatment-time F (8, 56) =3.481, P = 0.0025). Likewise, in probe trail (**B**) the crossing times and the percent time in the target quadrant by APP/PS1 mice, whether they received GA injection or not, were significantly lower compared to WT mice (one-way ANOVA, and LSD post hoc analysis; crossing time: APP/PS1+PBS vs WT mice F (2,12) =2.400, P = 0.0207; APP/PS1+GA vs WT mice F (2,12) =2.000, P = 0.0466; the percent time in the target quadrant: APP/PS1+PBS vs WT mice F (2,12) =13.28, P = 0.0284; APP/PS1+GA vs WT mice F (2,12) =14.42, P = 0.0180). (mean ± SEM, n = 5 per group, *P < 0.05, **P < 0.01, ****P < 0.0001).
